# Contextual sensory integration training vs. traditional vestibular rehabilitation: a pilot randomized controlled trial

**DOI:** 10.1186/s12984-023-01224-6

**Published:** 2023-08-12

**Authors:** Jennifer Kelly, Daphna Harel, Santosh Krishnamoorthy, Gene Fu, Brittani Morris, Andrew Medlin, Sarah Mischinati, Zhu Wang, John Sutera, Ken Perlin, Maura Cosetti, Anat V. Lubetzky

**Affiliations:** 1 Vestibular Rehabilitation, New York Eye and Ear Infirmary of Mount Sinai, New York, NY USA; 2 Department of Otolaryngology-Head and Neck Surgery, Icahn School of Medicine at Mount Sinai, New York, NY USA; 3 Department of Applied Statistics, Social Science and Humanities, Steinhardt School of Culture Education and Human Development, New York University, New York, NY USA; 4 Department of Physical Therapy, Steinhardt School of Culture, Education and Human Development, New York University, New York, NY USA; 5 Computer Science Department, New York University, Courant Institute of Mathematical Sciences, New York, NY USA

**Keywords:** Head mounted Display, HTC Vive, Balance, Vestibular Rehabilitation

## Abstract

**Background:**

We created a clinical virtual reality application for vestibular rehabilitation. Our app targets contextual sensory integration (C.S.I.) where patients are immersed in safe, increasingly challenging environments while practicing various tasks (e.g., turning, walking). The purpose of this pilot study was to establish the feasibility of a randomized controlled trial comparing C.S.I. training to traditional vestibular rehabilitation.

**Methods:**

Thirty patients with vestibular dysfunction completed the Dizziness Handicap Inventory (DHI), Activities-Specific Balance Confidence Scale (ABC), Visual Vertigo Analog Scale (VVAS), Functional Gait Assessment (FGA), Timed-Up-and-Go (TUG), and Four-Square Step Test (FSST). Following initial assessment, the patients were randomized into 8 weeks (once per week in clinic + home exercise program) of traditional vestibular rehabilitation or C.S.I. training. Six patients had to stop participation due to the covid-19 pandemic, 6 dropped out for other reasons (3 from each group). Ten patients in the traditional group and 8 in the C.S.I group completed the study. We applied an intention to treat analysis.

**Results:**

Following intervention, we observed a significant main effect of time with no main effect of group or group by time interaction for the DHI (mean difference − 18.703, 95% CI [-28.235, -9.172], p = 0.0002), ABC (8.556, [0.938, 16.174], p = 0.028), VVAS, (-13.603, [-25.634, -1.573], p = 0.027) and the FGA (6.405, [4.474, 8.335], p < 0.0001). No changes were observed for TUG and FSST.

**Conclusion:**

Patients’ symptoms and function improved following either vestibular rehabilitation method. C.S.I training appeared comparable but not superior to traditional rehabilitation.

**Trial registration:**

This study (NCT04268745) was registered on clincaltrials.gov and can be found at https://clinicaltrials.gov/ct2/show/NCT04268745.

**Supplementary Information:**

The online version contains supplementary material available at 10.1186/s12984-023-01224-6.

## Introduction

Vestibular disorders lead to complaints of dizziness, instability and falls [1]. Vestibular rehabilitation is an exercise-based intervention to address these complaints and improve function, postural instability and quality of life [2, 3]. The 2022 Clinical Practice Guidelines [2] (CPG) for vestibular rehabilitation noted that sufficient evidence suggests that vestibular exercises are effective compared with no intervention or placebo exercises. Given that, they noted that future research efforts should be directed to comparative effectiveness research with specific focus on the delivery of vestibular rehabilitation using technology. Research to identify the most effective methods of delivering vestibular rehabilitation and clarifying the role of technology, particularly immersive, portable virtual reality devices is a priority in the field [4]. Yardley and Redfern highlighted the need to combine rehabilitation for dizziness and multisensory integration with addressing the psychological needs of the patient [5]. This can be accomplished with virtual reality where exposure to complex environments happens in a safe manner under the supervision of a therapist, and the patient can leave the environment at any time [6]. Technology ranging from off-the-shelf games to laboratory large screens has been used for many years in vestibular rehabilitation and has been shown to be effective with regards to physical outcomes and for facilitating sensorimotor relearning for balance [7] though not necessarily better than traditional rehabilitation [8, 9]. Recent systematic reviews observed potential clinical benefits of virtual reality technology for vestibular rehabilitation compared with conventional vestibular rehabilitation based on low level of evidence [10, 11]. Yet they noted a gap in the literature related to utility of newer portable headsets and the efficacy of an individualized, context specific approach that can be used to train balance when the patient is standing and moving [10, 11].

These days, using virtual reality in a clinical setting to address multisensory integration is becoming increasingly accessible. Head Mounted Displays (HMDs: goggles that are worn on the head in lieu of a screen and projectors), such as the Oculus Rift and the HTC Vive, can potentially allow for a specific and individualized program, with minimal space requirements and a high level of immersion. The theoretical rationale supporting HMD programs for vestibular rehabilitation is clear [4]. HMDs can be used to generate a graded method for patients to experience semi-real environments in a non-threatening manner. The HTC Vive also allows for walking in a small space and newer untethered headsets will allow for free walking in a room. Nevertheless, while the technology is evolving rapidly, research regarding effectiveness and clinical application of new HMD’s is in its infancy. Micarelli et al. found that head movement training in sitting while participating in a driving game via the Oculus Rift was effective for patients with unilateral vestibular hypofunction when combined with a traditional vestibular program [12], with benefits maintained at 1-year follow up [13]. It is still currently unclear whether an individualized approach that allows for dynamic balance training with precise sensory exposure that matches the patients’ symptoms, imbalance and context-specific experience will show better benefit than other off-the-shelf games or laboratory technology for patients with vestibular disorders.

To begin answering these questions, we have created a clinical app using the HTC Vive headset to provide contextual sensory integration (C.S.I.) where patients work on their balance while being immersed in safe but increasingly challenging environment [14]. We have developed our app based on on-going feedback from patients regarding their daily participation restrictions and from physical therapists about the conditions they would want to be able to reproduce in the clinical setting (e.g., patients complaining about difficulty with patterned floors, complaining about crowded spaces). These specific environments are designed to mimic patients’ daily visual and auditory load that are not easily reproducible in traditional rehabilitation (i.e.: sounds, multidirectional visual flow, crowds, standing on the edge of a platform, maintaining balance when a train goes by, movements outside of base of support in response to an unexpected external stimulus, closed vs. open space). We previously showed that the app is feasible and was helpful in a small cohort of people with peripheral hypofunction treated in an outpatient vestibular clinic [15].

### Objectives

Our goal was to create an improved rehabilitation approach using immersive HMD technologies that is individualized to each patient’s functional complaints within the proper context. Low costs and simplicity of use increase the broader impact of this research and the possibility of a large-scale implementation in diverse clinical settings. The purpose of this specific pilot study was to develop the protocol and establish the feasibility of a randomized controlled trial (RCT) comparing C.S.I. training to traditional vestibular rehabilitation. To do that we compared functional and self-reported outcomes between groups before and after the intervention.

## Methods

This study including all covid modifications was approved by the BRANY institutional review board (IRB, # 19-02-223), the IRB at Icahn School of Medicine at Mount Sinai and New York University Committee on activities involving research subjects.

### Participants

All patients signed consent prior to enrolling in the study. We recruited patients referred to an outpatient vestibular rehabilitation clinic with chief complaints of dizziness and/or imbalance. The participants first underwent an initial vestibular physical therapy evaluation of approximately 60 min. The evaluation included: detailed history, oculomotor screening which included saccades, smooth pursuit and convergence, assessment of spontaneous and gaze evoked nystagmus with and without infrared goggles, Dix-Hallpike and roll test to rule out benign paroxysmal positional vertigo (BPPV), and bedside head impulse test to screen for vestibulo-ocular (VOR) impairment. The assessment also included gait speed and gait stability with head turns in both horizontal planes and vertical planes, as well as the modified clinical test of sensory integration to assess static balance. Peripheral vestibular hypofunction was diagnosed by either positive findings on bithermal caloric testing during videonystagmography and/or positive bedside head impulse test, head shaking nystagmus and gaze evoked nystagmus and/or clinical history characterized by sudden onset of vertigo lasting hours, aural symptoms unilaterally and ruling out other central causes [16, 17]. Central vestibular conditions were diagnosed based upon history of head injury leading to symptoms, history of resected acoustic neuroma or migraine history with episodic vertigo. See Table 1 for the breakdown of diagnoses. We excluded patients if they had bilateral or unstable vestibular loss or another neurological condition, active BPPV, acute orthopedic injuries, peripheral neuropathy and visual impairment not corrected with glasses.

**Table 1:** Description of Outcome Measures Collected at Baseline and Post

**Functional Outcomes**			
	**Description**	**Psychometric Properties**	**Comments**
Functional Gait Assessment (FGA)	Evaluates individuals on their ability to perform various motor tasks such as: walking with eyes closed, walking backwards, climbing stairs. There are 10 items, each is scored by a therapist on a scale of 0 (severe impairment) to 3 (normal).	Intraclass correlation coefficients of 0.86 and 0.74 were found for interrater and intra-rater reliability of the total FGA scores in vestibular disorders [36]. Internal consistency was 0.79 (no confidence intervals provided for either). The minimal clinically important difference (MCID) on the FGA is considered 4 points [19, 20]. A score lesser or equal to 22 / 30 indicates increased fall risk in community dwelling older adults [21].	An experienced vestibular physical therapist administered the FGA in this study.
Timed-Up and Go (TUG)	Patients are asked to rise up from a chair, walk at their comfortable speed 10 feet, turn around a cone, walk back and sit down. The faster performance out of two trials was recorded.	A score slower than 11.1 s in people with vestibular disorders [22] or slower than 13.5 s in community dwelling adults indicates increased fall risk [23]. An MCID of 3.4 s was established for patients post back surgery [24].	The TUG was administered by a research team member (physical therapist or physical therapy student).
The Four-Square Step Test (FSST)	A multidirectional stepping test of dynamic balance and coordination. Participants are asked to step over 4 canes on the floor in a clockwise and then counterclockwise direction while being timed. Patients did one practice trial and then we recorded the faster performance out of two trials.	A score > 15 s indicates increased fall risk in community dwelling adults over the age of 65 [25]. Whitney et al. identified a cut off score of 12 s for patients with vestibular disorders [26].	The FSST was administered by a research team member (physical therapist or physical therapy student).
**Self-reported Outcomes**			
Visual Vertigo Analog Scale (VVAS)	The participants mark the intensity of their dizziness on a scale of 0 to 10 cm in 9 situations of visual motions that typically provoke dizziness [27]. The score is calculated by measuring each item in centimeters, averaging the scores and multiplying by 10.	Symptom severity can be classified as none (0), mild (0.1 to 40), moderate (40–70) or severe (above 70) [28, 29].	
Activities Specific Balance Confidence Scale (ABC)	A subjective measure of confidence in performing activities without falling. Each item is scored from 0% (no confidence in one’s balance) to 100% (full confidence in one’s balance) [30].	A score of less than 67% indicates increased fall risk in community dwelling adults [31]. A minimal detectable change was identified as 13% in patients with Parkinson’s disease [32] and 14% in patients post stroke [33].	
The Dizziness Handicap Inventory (DHI)	The DHI has 25 items involving the functional, emotional, and physical domains. Each item is scored as ‘no’, ‘sometimes’ or ‘yes’ to evaluate self-perceived disability imposed by dizziness [30, 34].	The DHI is classified as mild (under 30), moderate (31–60) or severe (61–100) disability due to dizziness [22]. The MCID for the DHI is considered to be 18 points [34].	

### Procedure

When a clinician identified a patient as eligible, they reviewed the informed consent and explained the study procedures. Following consent, patients underwent a baseline assessment including functional measures (Functional Gait Assessment [FGA] [18–21], Timed-Up and Go [TUG] [22–24], The Four-Square Step Test [FSST]) [25, 26], and self-reported questionnaires (Visual Vertigo Analog Scale [VVAS] [27–29], Activities Specific Balance Confidence Scale [ABC] [30–33], The Dizziness Handicap Inventory [DHI] [30, 34]. See Table 2 for details. The assessment also included a postural control test using HMD. The full protocol and results of this assessment are reported elsewhere [35]. Briefly, standing hips-width apart on the floor, participants experienced two levels of visual surround and white noise while their head sway was recorded via the HTC Vive Pro headset. The total duration of the session was approximately 45 min. After the baseline assessment, the patients were randomized to 8 sessions of either traditional vestibular rehabilitation or C.S.I training followed by an immediate post assessment.

**Table 2:** Description of the C.S.I, Contextual Sensory Integration /Traditional Sample on Pre-treatment Variables

	Overall	Traditional	C.S.I.	P value
**Gender**	Female = 18 (60.00%) Male = 12 (40.00%)	Female = 9 (60.00%) Male = 6 (40.00%)	Female = 9 (60.00%) Male = 6 (40.00%)	P = 0.61##
**Head Thrust**	Abnormal = 12 (40.00%) Normal = 15 (50.00%) NT = 3 (10.00%)	Abnormal = 6 (40.00%) Normal = 9 (60.00%)	Abnormal = 6 (40.00%) Normal = 6 (40.00%) NT = 3 (20.00%)	P = 0.68##
**Head Shaking**	Abnormal = 11 (36.67%) Normal = 17 (56.67%) NT = 2 (6.67%)	Abnormal = 5 (33.33%) Normal = 10 (66.67%)	Abnormal = 6 (40.00%) Normal = 7 (46.67%) NT = 2 (13.33%)	P = 0.42##
**Gaze evoked nystagmus without fixation**	Abnormal = 8 (26.67%) Normal = 21 (70.00%) NT = 1 (3.33%)	Abnormal = 1 (6.67%) Normal = 14 (93.33%)	Abnormal = 7 (46.67%) Normal = 7 (46.67%) NT = 1 (6.67%)	P = 0.03##
**Presence of Migraine**	No = 19 (63.33%) Yes = 11 (36.67%)	No = 11 (73.33%) Yes = 4 (26.67%)	No = 8 (53.33%) Yes = 7 (46.67%)	P = 0.45##
**Age: in years mean (min, max, SD)**	46.97 (21, 78, 17.29)	48.6 (21, 78, 19.44)	45.33 (24, 70, 15.35)	P = 0.61#
**Onset in years: mean (min, max, SD)**	1.69 (0.08, 12, 2.94)	0.85 (0.08, 2.5, 0.76)	2.53 (0.08, 12, 3.98)	P = 0.39###
**Calorics via VNG, Videonystagmography (unilateral weakness = greater than 25%)**	Normal = 3 (10.00%) Weakness = 16 (53.33%) NT = 11 (36.67%)	Normal = 2 (13.33%) Weakness = 7 (46.67%) NT = 6 (40.00%)	Normal = 1 (6.67%) Weakness: 9 (60.00%) NT = 5 (33.33%)	P = 0.92##
**Diagnoses**	24/30 peripheral hypofunction 2 post-concussion 2 vestibular migraine 2 acoustic neuroma	11 peripheral hypofunction 1 post-concussion 2 vestibular migraine 1 acoustic neuroma	13 peripheral hypofunction 1 post-concussion 0 vestibular migraine 1 acoustic neuroma	

#: One way ANOVA, Analysis of Variance; ##: Chi-square for proportions; ###: Kruskal-Wallis

NT: Not Tested

### Randomization and group allocation

We used a blocked randomization method. Instead of randomizing each patient individually, this scheme randomizes several patients at a time in such a way as to ensure that equal numbers are allocated to each group across each segment of time during the length of the study. For example, if the block size is four, we randomize four patients at a time ensuring that two patients are allocated to the C.S.I group and two patients to the traditional group. As it happens, there are six different possible ways we could randomize four patients equally to two treatments. The randomization was done following the baseline assessment and only the study statistician had access to the randomization sequence.

### Interventions

Following the baseline assessments, we randomized patients to a C.S.I. group or a traditional vestibular rehabilitation control group. We planned each program to be 8 weeks (1 30-minute weekly session + home program). We conducted a post-assessment, identical to the baseline assessment, within one week from the completion of the 8th intervention session.

A detailed description of a single patient intervention and home exercise program can be found in appendix A (C.S.I.) and B (Traditional). Below we provide an overview of possible variations. The main difference between participants was the timing of progression which was individualized based on patient symptoms (dizziness and / or instability). Each exercise (in clinic or home) was prescribed at the highest level of challenge that was considered safe (i.e., no loss of balance or no significant increase in dizziness). We assigned all patients a home exercise program (Appendices A & B, the C.S.I group had similar exercises without eyes closed tasks, effectively 2 min less exercise per day) which they were asked to complete twice daily, for 5 to 10 min. Their home program consisted of gait, gaze stability, and static balance activities [2].

For patients in the C.S.I. group (Appendix A) the progression of environments started with the most salient to the patient and this varied from patient to patient and eventually most patients completed several different environments (e.g., street, subway, airport). The duration varied with starting point at 1 min with increase in time up to 5 min based upon patients’ symptoms. Beyond duration, the exact progression the patient underwent varied by therapist. Some therapists chose to change the complexity of the scene (i.e., increased amount people, increased speed of people adding sounds, or changing directions of walking), while others chose to add tasks in the scene (i.e., walking for a maximum. of 4–5 steps, changing base of support or adding head turns). For a video demonstration of the C.S.I app, see Supplemental Videos 1 & 2.

The following are the exercise variation for the traditional group (Appendix B): **Gait**: walking with head turns, progress with range, speed and planes of head movement; change of walking base of support: wide, normal, tandem; **Gaze**: focus on a target while moving head side to side / up down. Progress with speed, duration, busier background, standing to walking; **Balance**: standing balance tasks, progress with BOS (wide to narrow to tandem), support surface, eyes closed, duration, head turns.

### Statistical analysis

We compared the sample before and after shut-down due to covid-19 as well as those who dropped out compared to those who did not using independent sample t-tests for continuous measures that were normally distributed, a Kruskal-Wallis test for skewed continuous variables, and a chi-square test for proportions.

To investigate the effect of the intervention, we fit a linear mixed effects model for each outcome measure of interest (FGA, DHI, VVAS, ABC, TUG, FSST) on group (C.S.I. or Traditional), time (pre-, post-intervention) and their interaction. The models also included random intercepts for each participant to account for the inherent correlation between each participant’s performances across the two timepoints. We used sum coding for the categorical predictor variables (group and time) in order to obtain estimates for average differences. Therefore, the coefficient for the factor of time can be interpreted as the average changes in time across both groups. The interaction term between the two variables can be interpreted as any differences observed in one group but not in the other.

Because we did not observe any significant differences in the baseline outcome measures between those who participated in the entire study and those who dropped out (see Appendices C & D), we conducted these analyses using intention to treat through the linear mixed effects model. Specifically, the linear mixed effects model uses all available data to obtain estimates of the population averages for each group without more advanced imputation strategies (such as multiple imputation). Figures and analysis were done in R version 4.1.2 (2021-11-01, The R project for Statistical Computing).

## Results

### Sample

**Fig. 1:**
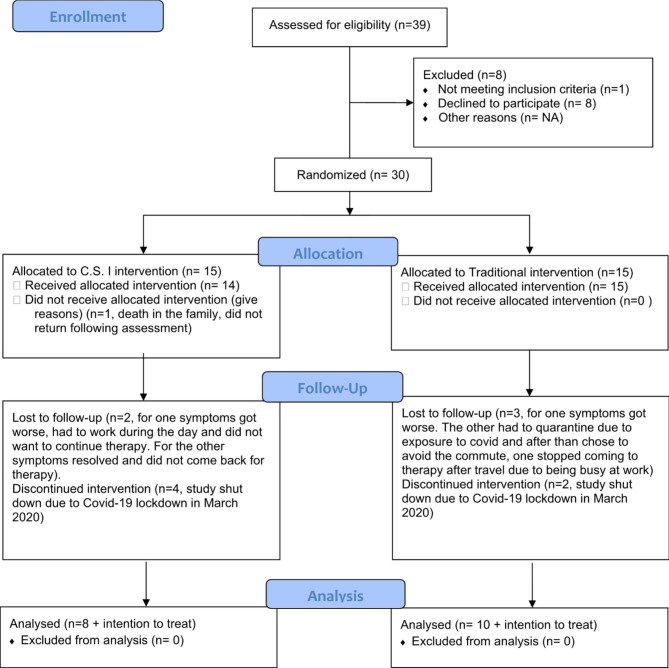
Recruitment flow diagram

We began recruitment in September 2019, it was shut down in March 2020 due to the covid-19 pandemic, and resumed in September 2020. See Fig. 1 for the flow of recruitment and Table 1 for description of the sample. There were no observed differences between patients before and after the covid shutdown (Appendix C), no observed differences between patients who dropped out or completed and no observed differences between group in attrition % (Appendix D). Due to covid-19 constraints, such as quarantines due to exposure, the average time between pre and post assessment was 11 weeks (SD = 2.69).

### Effect of intervention

**Fig. 2:**
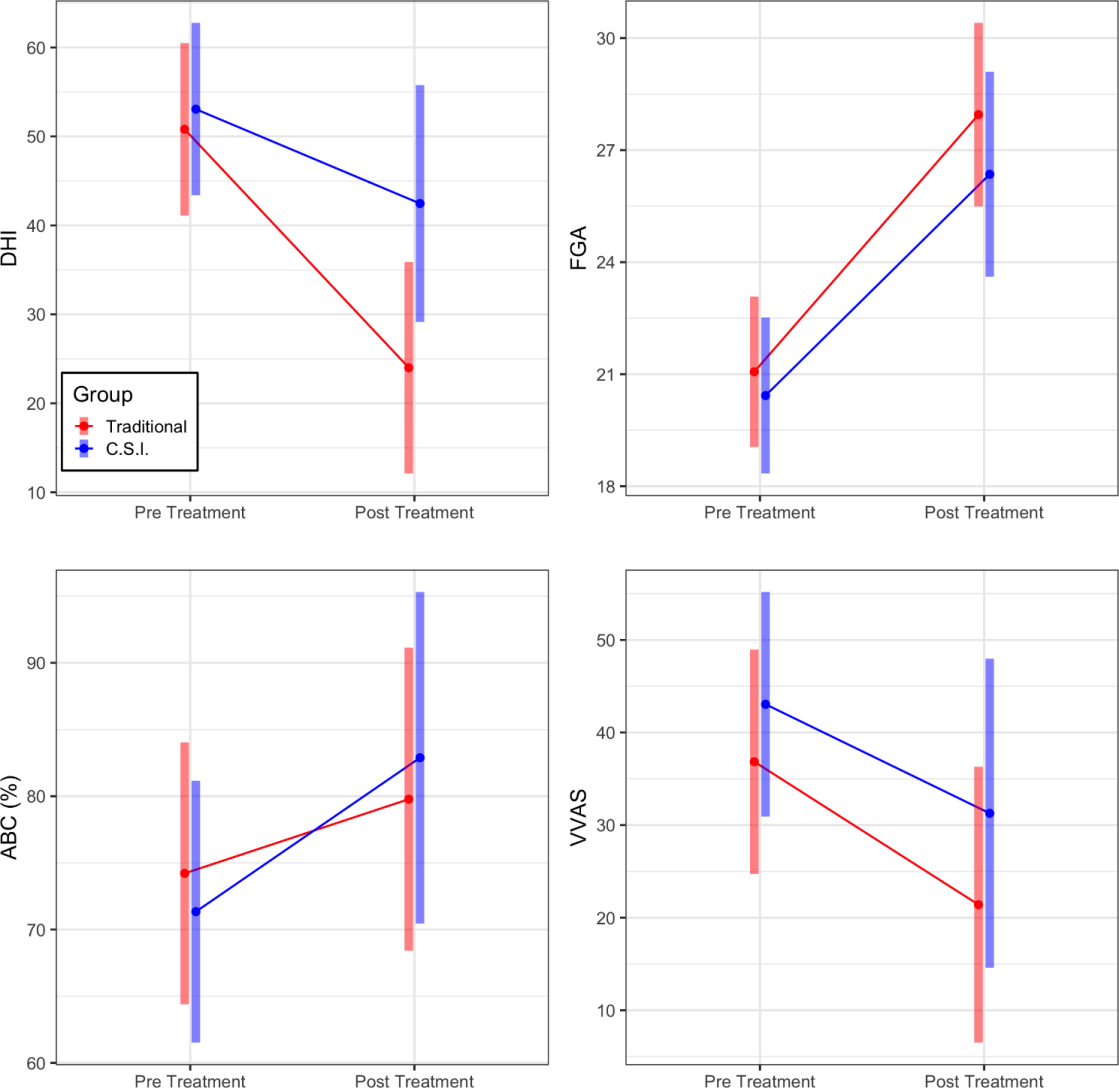
Pre and Post estimated marginal mean and their respective 95% confidence intervals for the traditional and C.S.I groups on outcomes that showed a significant change over time in both groups: The Dizziness Handicap Inventory (DHI pre: Traditional 50.8 [41.1, 60.49], C.S.I 53.07 [43.37, 62.76]. post: Traditional 24 [12.11, 35.89], C.S.I 42.4 [29.15, 55.77]); Functional Gait Analysis (FGA pre: Traditional 21.07 [19.05, 23.08], C.S.I 20.43 [18.34, 22.52]. post: Traditional 27.95 [25.5, 30.41], C.S.I 26.35 [23.61,29.1]); Activities Specific Balance Confidence Scale (ABC pre: Traditional 74.21% [64.39, 84.03], C.S.I 71.34% [61.52, 81.16]. post: Traditional 79.78% [68.41, 91.14], C.S.I 82.89% [70.46, 95.31]); Visual Vertigo Analog Scale (VVAS, pre: Traditional 36.84 [24.72, 48.97], C.S.I 43.05 [30.93, 55.17]. post: Traditional 21.41 [6.51, 36.31], C.S.I 31.28 [14.59, 47.97])

**Fig. 3:**
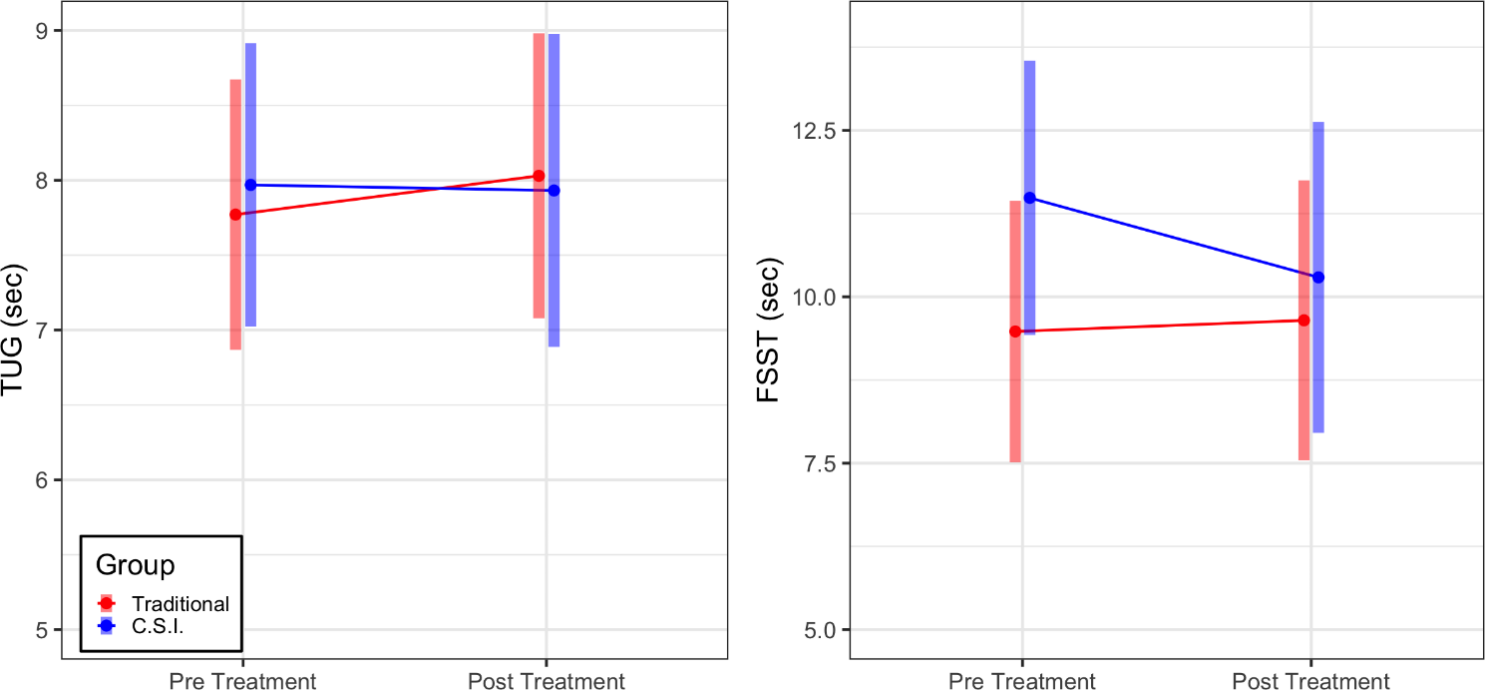
Pre and Post estimated marginal mean and their respective 95% confidence intervals for the for the traditional and C.S.I groups on outcomes that did not change over time in either group: Timed-Up and Go (TUG, pre: Traditional: 7.77 s [6.87, 8.67], C.S.I: 7.97 s [7.02, 8.92], post: Traditional: 8.03 s [7.08, 8.98], C.S.I: 7.93 s [6.88, 8.98]); The Four-Step Square Test (FSST pre: Traditional 9.48 s [7.52, 11.44], C.S.I 11.49 s [9.43, 13.55 ] post: Traditional 9.65 s [7.54, 11.75], C.S.I 10.29 s [7.96, 12.63])

We observed a significant main effect of time for 4 outcome measures (Fig. 2). For the DHI, there was a mean difference of -18.703 (95% CI [-28.235, -9.172], p = 0.0002, meeting the MCID). Six out of 10 participants in the traditional group exceeded the DHI MCID, 9/10 reported some improvement. In the C.S.I group, these numbers were 2/8 and 5/8 respectively. For the ABC there was a mean difference of 8.556 (95% CI [0.938, 16.174], p = 0.028). Two out of 10 participants in the traditional group exceeded 13% improvement, 4/10 reported some improvement. In the C.S.I group these numbers were 3/8 and 5/8 respectively. For the VVAS, there was a mean difference of -13.603 (95% CI [-25.634, -1.573], p = 0.027). Five of 10 participants in the traditional group and 7/8 participants in the C.S.I group reported some improvement. For the FGA, there was a mean difference of 6.405 (95% CI [4.474, 8.335], p < 0.0001, exceeding the MCID). Eight out of 10 participants in the traditional group and 6 out of 8 participants in the C.S.I group exceeded the FGA MCID. Further, pre intervention the traditional group had 6 at fall risk and the C.S.I group had 7. At the end, these numbers were 0 and 1 respectively. The 1 patient who scored 21 started the study at 16. For all these measures, there were no significant differences between groups, nor a significant group by time interaction. Furthermore, there was no evidence of a difference across time, group, nor their interaction for the TUG and FSST (all p > 0.05, Fig. 3).

## Discussion

In this pilot randomized trial patients demonstrated significant, clinically important differences over time in both groups with no evidence of significant differences between groups. Specifically on the FGA both groups began on average at fall risk (below 22) and came closer to maximal score (on average 28 or 26 points out of 30) following the intervention. These changes of a little over 20% are similar to the ones reported in a recent systematic review [11]. There was a strong theoretical rationale to believe that an individualized, context-specific approach that allows for immersive training in standing and stepping would provide greater benefits for patients with vestibular dysfunction than off-the-shelf games. Nevertheless, our findings are similar to Meldrum et al.,[8] who compared Wii fit exercises to traditional vestibular rehabilitation, and found that both groups improved with no significant difference between groups. Likewise, training within a virtual grocery store projected on a large screen led to similar functional and self-reported changes as traditional training in a group of patients with vestibular disorders [1]. The low cost and portability of the HTC Vive is expected to allow for smoother clinical translation. Indeed, Micarelli et al., [12] studied 2 groups: one who received HMD therapy at home and one who did not, both groups received in clinic therapy for 8 sessions. The HMD group showed larger improvements but differences in total therapy time could have contributed to the difference. In addition, the HMD training was done in sitting. Our study adds to the body of literature supporting the effectiveness of vestibular rehabilitation to improve dynamic balance, balance confidence and reduce dizziness in patients with vestibular disorders. Because participants in both groups were considered chronic (symptoms lasting 3 months or longer at the beginning of the study) except for 1 patient in each group who were at the subacute phase, it is unlikely that natural recovery influenced the results [2]. Below we discuss potential explanations to our findings and suggestions for future research to promote vestibular rehabilitation.

C.S.I. is a viable and good intervention option that patients enjoyed with limited symptoms that is not expensive and is feasible in a clinical setting. We designed the app with free graphics that can create sensory load without requiring high computational power [15]. Theoretically, hyper-real simulations may facilitate better transfer to real-life skills [37], however this notion has not been supported with human data [38]. Currently, the computational power required for hyper-real simulations will increase costs, may lead to cybersickness associated with latency and lags [39] and could delay clinical adoption. That being said, the HMD industry continues to evolve rapidly, and it is possible that in the near future hyper-real simulations will become more affordable as well. It would be then necessary to compare the effect of abstract, simple visuals to more precise simulations.

While we did not formally measure enjoyment, most participants were interested in the study because virtual reality was offered and patients in the C.S.I. group appreciated the engagements in salient environments. Greater enjoyment in computerized interventions has been reported before [8]. Indeed, our small pilot study, as well as the work of others mentioned above, indicates a need for further investment in virtual reality training for vestibular dysfunction particularly if the field can advance towards making this a portable and viable option for in-home training as well as in more clinics around the world. In the post-covid era, there is a great need for technology that will create engaging and effective remote options for patients especially with the possibility for remote monitoring. Note, however, that currently there is no evidence that virtual reality interventions are better than traditional vestibular rehabilitation.

The two interventions in this study were inherently different, i.e., context specific training (salient balance challenges with dynamic visual load) versus traditional vestibular rehab (gait training and gaze stability exercises), yet the results were remarkably similar. That brings the question of whether the more important aspect to therapy is the home exercise program, including vestibular-specific exercises regardless of method of delivery (i.e. head turning, walking, visual overload). Note that the only difference between the home exercise programs was that the C.S.I group did not perform exercises with eyes closed. It is also possible that research creates a selection bias where patients who agree to be involved in research tend to be more motivated, which could be key to improvement. At the conclusion of our study, the question remains regarding the importance of incorporating different contexts in balance training. More research needs to be done regarding the importance of context in vestibular rehabilitation and what crucial features in virtual reality may help patients the most. In addition, our app allowed for a complete freedom in clinical decision making. The therapists could choose how and when to progress based on patients’ subjective response. Broad implementation of virtual reality technology could allow future studies to incorporate machine learning approaches and potentially design a progression algorithm and test whether such algorithm could enhance patients’ outcomes.

We used functional outcome measures that are recommended by the APTA Vestibular Edge Taskforce [40]. The TUG and FSST have consistently been shown to be useful in addressing fall risk in the vestibular population [26], but in the current study patients generally started at a good level of performance (well below the 11 s cutoff) and therefore it is unsurprising that no changes were observed. The FGA, on the other hand, while taking longer to implement (10–15 min vs. 10–15 s) and requires clinicians’ expertise and judgment, showed excellent sensitivity to change with both groups improving from below to above the fall risk cutoff. The TUG and FSST in this study were administered by a research team member who was a licensed physical therapist or physical therapy student and while it is possible that there were errors in administration, we standardized the verbal instructions and trained the team on tests administration prior to the study. We also do not believe than any errors would occur more frequently in one arm of the study versus the other. It is also possible that people in New York City generally walk faster than the national norms. Pre rehabilitation the average TUG scores were about 8 s in both groups. For comparison, the literature suggests that a score slower than 11.1 s in people with vestibular disorders [22] or slower than 13.5 s in community dwelling adults indicates increased fall risk [23]. Only 1 patient in the entire sample scored over 10 s pre and post rehabilitation (10.82 s pre, 10.32 s post). Future studies should investigate whether the TUG and FSST should continue to be evaluated as an outcome measure in this population or whether additional norms should be established for urban areas.

Moderate to weak evidence from the clinical practice guidelines for peripheral hypofunction recommend 5–7 weeks of vestibular rehabilitation once a week [2]. Most studies of vestibular rehabilitation programs have a duration of 4 weeks to 9 weeks and we chose an 8 week program to reflect common practice. We observed a significant decrease in subjective report of symptoms, however, most patients still reported mild to moderate dizziness after 8 visits indicating the possible need for continued therapy beyond this timeframe. It is also possible that weekly frequency greater than once per week would lead to further improvements. Indeed, some of our patients continued traditional rehabilitation after the completion of the study. Clinicians may need to extend the number of sessions based upon individual patients’ complaints and the field could benefit from biomarkers that support these decisions and suggest the potential for continued recovery.

Recent CPG guidelines state that clinicians may prescribe static and dynamic balance exercises for a minimum of 20 min for at least 4 to 6 weeks although the strength of the recommendation is weak [2]. In the current study we prescribed 10 to 20 min daily of home exercises to both groups, which included both static and dynamic balance activities and gaze stability. This is significantly less than the recommendation and yet the outcomes improved similar to other studies. The CPG recommend that performing gaze stabilization exercises 3 times per day for a total of 20 min daily for 6 weeks may be sufficient to induce recovery of dynamic visual acuity (DVA) in individuals with chronic unilateral vestibular hypofunction. Though we did not specifically look at recovery of DVA through clinical DVA testing or Video Head Impulse Test (vHIT), our patients did perform gaze stability exercises daily for a range of 4 to 8 min. Implementing pre and post evaluation of covert and overt saccades via vHIT in future studies would be beneficial to determine if lower exercise dosage with or without virtual reality training can lead to changes in gaze stability.

### Limitations

The study was designed as a small pilot RCT and then became even smaller due to the challenges posed to in-person research in an outpatient setting by the covid-19 pandemic. The high dropout rate (both due to the study closure in March and later associated with challenges in transportation and quarantines), while similar between groups, may have influenced the outcome. While both groups showed significant improvements, it is possible that we were under-powered to detect differences between groups. It is possible for example, that the C.S.I intervention could lead to greater gains in visual vertigo (87.5% of the C.S.I group reported improvement vs. 50% of the traditional) and the traditional intervention in overall disability due to dizziness (62.5% reported some improvement in the C.S.I group vs. 90% of the traditional) but this needs to be investigated in future, larger studies. Functionally, however, the groups showed no trend for differences between them. This was also seen in our companion analysis of head sway data pre and post intervention where the vestibular participants were significantly higher than controls on all outcomes pre rehabilitation. Post rehabilitation they were only significantly higher on sway in mid-frequencies (0.25 to 0.5 Hz) with no indication of any difference between the intervention groups [35].

We did not officially track adherence to the home program and no long-term follow up was conducted. In order to continue the study under covid restrictions including limited personnel, quarantines, difficulty of patients to travel etc., we had to conduct several protocol changes that may influence the internal and external validity of the study. We originally planned to recruit only patients with chronic unilateral peripheral hypofunction. To maximize recruitment post covid we included patients with central disorders as well (2 post-concussion, 2 vestibular migraine) as well as 2 patients (1 in each group) who were considered subacute. While this shows that the C.S.I. intervention is feasible in patients with central disorders, the sample is too small for generalizability. The program was originally planned for 10 weeks: baseline, 8 sessions, post sessions. Due to quarantines and difficulty with travel, some patients took longer to complete, and we often opted to run the assessment on the last day of rehabilitation to mitigate the risk that a patient may not come back due to exposure to the virus. We had originally planned on having a blinded assessor to complete the FGA pre and post but following covid this was no longer feasible, and the treating therapist had to conduct the FGA which may create bias. Note that these modifications influenced both groups in a similar way. While theoretically, all outcomes could be influenced by an assessor, the FGA requires greater clinical judgment than stopwatch-based measures or self-reported outcomes that patients complete on their own. The lack of a blinded outcomes assessor may have influenced the result. There was one variable that significantly differed between groups at baseline: more patients in the C.S.I. group presented with gaze evoked nystagmus which is typically indicative of a more acute lesion. However, these patients were on average equally chronic as the traditional group and so the likelihood of this one different finding influencing the study outcome is low. Lastly, HMD technology continues to develop rapidly, and untethered, high-end headsets are already commercially available at low cost. While this technology carries huge potential for patient-specific sensory integration training in the clinic and possibly at the home, it is important to consider that the hardware was not originally developed for vestibular patients and so development of vestibular-specific applications and rigorous research regarding safety, benefits, progression and regression rules etc. is required to support this potential clinical translation and make sure that only the most effective and safe interventions are disseminated on a large scale.

## Conclusions

In this pilot randomized clinical trial, patients with vestibular disorders showed clinically important improvements following 8 weeks of vestibular rehabilitation regardless of the intervention approach: traditional vestibular program or contextual sensory integration program via a vestibular-specific HTC Vive application. HMD training within increasingly complex immersive environments appears to be a promising adjunct modality for vestibular rehabilitation but currently does not appear to be superior to other approaches. Our results need to be interpreted with caution because our study is limited by a small and diverse sample. A future larger study with a long-term follow up is required prior to applying these results clinically.

### Electronic supplementary material

Below is the link to the electronic supplementary material.

Supplementary Material 1: Supplementary Video 1. Demonstration of training in the subway scene.

Supplementary Material 2: Supplementary Video 2. Demonstration of training in the airport scene.

Supplementary Material 3: Appendix A: Weekly progression for a single patient in the C.S.I program, 8 sessions over 9 weeks. Appendix B: Weekly progression for a patient in the Traditional program, 8 sessions over 8 weeks. Appendix C: Description of the Before/After covid Sample. Appendix D: Description of the Sample Based on Drop Out Status.

Supplementary Material 4

## Data Availability

The datasets for the current study are available from the corresponding author upon request.

## Abbreviations

HMD: Head Mounted Display
C.S.I: Contextual Sensory Integration
ABC: Activities-specific Balance Confidence scale
DHI: Dizziness Handicap Inventory
VVAS: Visual Vertigo Analog Scale
FGA: Functional Gait Analysis
FSST: Four Square Step Test
TUG: Timed-Up and Go test
DVA: Dynamic Visual Acuity
vHIT: Video Head Impulse Tests
