# Supplementary material for: Contextual sensory integration training vs. traditional vestibular rehabilitation: a pilot randomized controlled trial
Source: J Neuroeng Rehabil. 2023 Aug 12;20:104. doi: 10.1186/s12984-023-01224-6 (PMC10422780; doi:10.1186/s12984-023-01224-6)
Supplement: Supplementary file 3 — Supplementary Material 3: Appendix A: Weekly progression for a single patient in the C.S.I program, 8 sessions over 9 weeks. Appendix B: Weekly progression for a patient in the Traditional program, 8 sessions over 8 weeks. Appendix C: Description of the Before/After covid Sample. Appendix D: Description of the Sample Based on Drop Out Status. [file 12984_2023_1224_MOESM3_ESM.docx]

Appendix A: Weekly progression for a single patient in the C.S.I program, 8 sessions over 9 weeks

| Session | Exercise performed | SSQ | Patient comments | Home Exercise Program |
| --- | --- | --- | --- | --- |
| 1 | City x 3 minutes, minimal amount of people and speed  City x 2 minutes, same  Subway x 2 minutes, minimal amount of people and speed  Subway x 3 minutes, increased amount of people and speed | Pre: 4  Post: 4 | Before session: “symptoms are intermittent”  In session: “closed scene bothers me more” | Walking with head turns center/right, center/left, center/up and center/down every 3 steps  VOR x 1, vertical and horizontal x 60 seconds |
| 2 | City x 3 minutes, minimal amount of people and speed  City x 3 minutes, same  Airport x 3 minutes, minimal amount of people and speed  Airport x 3 minutes, increased amount of people and speed | Pre: 7  Post: 5 | Before: “dizzy 24 hours after last time”  During: “open is better” “less dizzy after airport” | Walking with head turns side to side, up and down every 3 steps  VOR, same |
| 3 | City x 4 minutes, minimal amount of people and speed, added head movement  City x 4 minutes, increased amount of people and speed  Subway x 4 minutes, minimal amount of people and speed  Subway x 4 minutes, increased amount of people and speed  Airport x 4 minute, same, added patterned floor | Pre: 4  Post: 3 | Before: “intensity of dizziness has decreased, happy” | Same, no progression |
| 4 | ** first time added sound, level 1  City x 3 minutes, minimal amount of people and speed  Airport x 3 minutes, increased amount of people and speed  Airport x 3 minutes, moderate amount of people and increased sound level to 2  Subway x 3 minutes, moderate amount of people and speed | Pre: 4  Post: 5 | Before: “went weekend without dizziness”  During: “sound is more difficult” | Walking, same  VOR, increased to 2 minutes |
| 5 | ** sound on level 2  City x 3 minutes, moderate amount of people and speed  City x 3 minutes, increased amount of people and speed  Airport x 3 minutes, moderate amount of people and speed  Airport x 3 minutes, same  Subway x 3 minutes, moderate amount of people and speed | Pre:8  Post: 4 | Before: “had set back with increased dizziness” | Same, no progression |
| 6 | ** sound on level 2  City x 3 minutes, moderate amount of people and speed  City x 3 minutes, increased amount of people  Subway x 3 minutes, moderate amount of people and speed  Subway x 3 minutes, same | Pre:2  Post: 1 | Before: “balance better” “continues to have head fullness” | Walking with head turns in diagonal planes every 3 steps  VOR same |
| 7 | ** all with sounds at highest and head movement, turning around, walking  City x 4 minutes, moderate amount of people and speed  Airport x 4 minutes, increased amount of people and speed  Airport x 4 minutes, same  Subway x 4 minutes, moderate amount of people and speed | Pre: 0  Post: 1 | Before: “minimal dizziness and not thinking about it”  During: “overstimulated with the airport” | Same, no progression |
| 8 | ** all with highest level of sound and head movement, turning and walking  City x 5 minutes, moderate amount or people and speed  Subway x 5 minutes, moderate amount of people and speed  Airport x 5 minutes, increased amount of people and speed | Pre: 1  Post: 1 | Before: “dizziness almost gone” | Same no progression |

Appendix B: Weekly progression for a patient in the Traditional program, 8 sessions over 8 weeks

| Session | Exercise performed | SSQ | Comments | Home Exercise Program |
| --- | --- | --- | --- | --- |
| 1 | Static Balance:  REC, ¼ SREC, ½ SREC x 1 minute  Gait x 50 feet:  With head turns side to side every 3 steps  With head turns up and down every 3 steps, then every 2 steps  Gaze stability:  HVOR 1 x 1 minute  VVOR 1 x 1 minute | Pre:7  Post:16 | “Symptoms are getting better” | ½ SREC x 1 minute  Walking with head turns up and down every 2 steps, side to side every 3 steps |
| 2 | Static Balance:  ½ SREC x 1 minute  ¾ SREC x 1 minute  Gait x 50 feet:  Walking with head turns up and down, side to side every 3 steps  Walking with head turns in diagonal planes every 3 steps  Tandem gait x 25 feet  Gaze stability:  HVOR 1 x 1 minute  VVOR 1 x 1 minute | Pre:6  Post: 6 | “I’m doing my home exercise program and it is getting easier” | ¾ SREC x 1 minute  Walking with head turns in diagonal planes every 3 steps  HVOR, VVOR x 1 minute each |
| 3 | Static Balance:  ¾ SREC x 1 minute  Gait x 50 feet:  Walking with head turns side to side, up and down and diagonal every 3 steps  Tandem walking x 50 feet no head turns  Gaze stability:  HVOR 1 x 1 minute  VVOR 1 x 1 minutes | Pre: 6  Post: 6 | “Being tired makes my symptoms worse” | Same as session |
| 4 | Static Balance:  7/8 SREC x 1 minute  Gait x 50 feet:  Walking forwards with head turns in multi planes every 3 steps x 2 reps  Tandem walking with head turns side to side every 3 steps x 50 feet  Gaze stability:  HVOR 1 with checkboard background x 1 minute  VVOR I (same as HVOR) | Pre: 5  Post: 6 | “Feeling much better than before but not 100% complaint with HEP” | 7/8 SREC x 1 minute  Walking with head turns in multi-planes forward every 3 steps  Gaze stability as in session |
| 5 | Static Balance:  7/8 SREC x 1 minute  SREC X 1 minute but had LOB  Gait x 50 feet  Walking with head turns forwards and backwards with head turns in multi-planes every 3 steps  Gaze stability:  HVOR 1 with checkboard background x 1 minute  VVOR 1(same as HVOR) | Pre:5  Post:5 | “I don’t feel like the floor is shifting anymore” | 7/8 SREC x 1 minute  Walking with head turns forwards in multi-planes every 3 steps  HVOR / VVOR I with checkboard x 1 minute |
| 6 | Static Balance:  7/8 SREC x 1 minute  Gait x 50 feet:  Walking forward and backwards with head turns in multi-planes every 3 steps x 2  Tandem walking 50 feet with head turns side to side every 3 steps  HVOR 2 x 1 minute  VVOR 2 x 1 minute | Pre:6  Post: 8 | “I get symptoms when I’m tired” | Same as session |
| 7 | Static Balance:  SREC x 1 minute  Gait x 50 feet:  Walking with head turns forwards and backwards in multi planes every 3 steps x 2  Gaze stability:  HVOR 2 x 1 minute  VVOR 2 x 1 minute | Pre: 6  Post: 2 | “Head feeling much better” | Same as session |
| 8 | Static Balance: SREC 60 seconds  Gait x 50 feet:  walking forwards and backwards with multi-plane head turns every 3 steps x 2  Tandem walking EO/EC 3:1 50 feet  Gaze stability:  HVOR 2 x 1 minute  VVOR 2 x 1 minute | Pre: 4  Post: 4 | “Feeling much better” | No progression |

REC, Romberg Eyes Closed (feet together); SREC, Sharpened Romberg Eyes Closed; HVOR, Horizontal Vestibular Ocular Reflex; VVOR, Vertical Vestibular Ocular Reflex; EO/EC, Eyes Open / Eyes Closed

Appendix C: Description of the Before/After covid Sample

|  | Overall | Before-covid | After-covid | P-Value |
| --- | --- | --- | --- | --- |
| Gender | Female = 18 (60.00%)  Male = 12 (40.00%) | Female = 8 (88.89%)  Male = 1 (11.11%) | Female = 10 (47.62%)  Male = 11 (52.38%) | P = 0## |
| Age: mean (min, max, SD) | 46.97 (21, 78, 17.29) | 51.11 (23, 78, 19.39) | 45.19 (21, 76, 16.49) | P = 0.4# |
| ABC: mean (min, max SD) | 72.78 (24.38, 100, 17.91) | 74.63 (32.5, 100, 21.5) | 71.98 (24.38, 94.38, 16.67) | P = 0.72# |
| VVAS: mean (min, max, SD) | 39.95 (0.77, 88.89, 21.69) | 34.01 (14.44, 60, 12.97) | 42.49 (0.77, 88.89, 24.33) | P = 0.23# |
| DHI: mean (min, max, SD) | 51.93 (24, 90, 17.68) | 47.56 (32, 74, 12.16) | 53.81 (24, 90, 19.53) | P = 0.38# |
| FGA: mean (min, max, SD) | 20.76 (13, 29, 4.36) | 21.22 (13, 29, 6.38) | 20.55 (15, 29, 3.28) | P = 0.77# |
| Onset__years: mean (min, max, SD) | 1.69 (0.08, 12, 2.94) | 0.82 (0.17, 2.5, 0.84) | 2.06 (0.08, 12, 3.43) | P = 0.4### |

*Note:*

#: One way ANOVA; ##: Chi-square for proportions; ###: Kruskal-Wallis

Appendix D: Description of the Sample Based on Drop Out Status

|  | Overall | Did not drop out | Dropped out | P-Value |
| --- | --- | --- | --- | --- |
| Gender | Female = 18 (60.00%)  Male = 12 (40.00%) | Female = 16 (66.67%)  Male = 8 (33.33%) | Female = 2 (33.33%)  Male = 4 (66.67%) | P = 0.23## |
| Age: mean (min, max, SD) | 46.97 (21, 78, 17.29) | 49.62 (21, 78, 17.47) | 36.33 (23, 52, 12.69) | P = 0.09# |
| ABC: mean (min, max, SD) | 72.78 (24.38, 100, 17.91) | 72.38 (24.38, 100, 19.33) | 74.36 (63.13, 88.88, 11.75) | P = 0.81# |
| VVAS: mean (min, max, SD) | 39.95 (0.77, 88.89, 21.69) | 38.59 (0.77, 88.89, 23.73) | 45.37 (36.67, 63.33, 9.65) | P = 0.29# |
| DHI: mean (min, max, SD) | 51.93 (24, 90, 17.68) | 50.33 (24, 90, 18.9) | 58.33 (46, 74, 10.39) | P = 0.33# |
| FGA: mean (min, max, SD) | 20.76 (13, 29, 4.36) | 20.5 (13, 29, 4.32) | 22 (16, 29, 4.85) | P = 0.49# |
| Onset__years: mean (min, max, SD) | 1.69 (0.08, 12, 2.94) | 1.28 (0.08, 12, 2.38) | 3.32 (0.17, 12, 4.48) | P = 0.31### |

*Note:*

#: One way ANOVA; ##: Chi-square for proportions; ###: Kruskal-Wallis
