# Supplementary material for: Contextual sensory integration training vs. traditional vestibular rehabilitation: a pilot randomized controlled trial
Source: J Neuroeng Rehabil. 2023 Aug 12;20:104. doi: 10.1186/s12984-023-01224-6 (PMC10422780; doi:10.1186/s12984-023-01224-6)
Supplement: Supplementary file 4 — Supplementary Material 4 [file 12984_2023_1224_MOESM4_ESM.docx]

I included links to Video appendices 1 and 2 in case the mp4 files did not upload properly:

[Video 1 Subway](https://drive.google.com/file/d/1l_bYDAGmyYzDjrmbAlabj1BYpr-llUmP/view?usp=sharing)

[Video 2 Airport](https://drive.google.com/file/d/1c1lsKA2whKyyeRTOR4JMcAmhjnfTyOn7/view?usp=sharing)
